# Neurological and immunological dysfunction in two patients with *Bartonella henselae* bacteremia

**DOI:** 10.1002/ccr3.977

**Published:** 2017-04-26

**Authors:** David L. Kaufman, Andreas M. Kogelnik, Robert B. Mozayeni, Natalie A. Cherry, Edward B. Breitschwerdt

**Affiliations:** ^1^ Open Medicine InstituteMountain ViewCalifornia; ^2^ Galaxy DiagnosticsResearch Triangle ParkNorth Carolina; ^3^ Department of Clinical Sciences and the Intracellular Pathogens Research LaboratoryInstitute for Comparative MedicineCollege of Veterinary MedicineNorth Carolina State UniversityRaleighNorth Carolina

**Keywords:** *Bartonella*, immune dysfunction, Infection, neurological symptoms, vectorborne

## Abstract

Recently, BAPGM enrichment culture has documented *Bartonella* bacteremia in previously healthy, “nonimmunocompromised” patients following arthropod exposures. Neurobartonellosis should be among the differential diagnoses for patients with persistent or recurrent neurological symptoms of undetermined etiology. Microbiological and immunological testing should be concurrently pursued to determine whether defective immune function accompanies *Bartonella* bacteremia.

## Case Reports

### Patient 1

A 49‐year‐old female veterinarian from California was previously healthy working 60 h a week managing a cat shelter with 500 cats. These factors may have predisposed acquisition of *B. henselae* and failure to immunologically eliminate the infection. In October 2011, she experienced an extensive infestation of mange mites (*Sarcoptes scabiei* or *Notoedres cati*) after handling an infested cat. One month later, she experienced an episode of confusion and severe headache. There was no prior history of headaches or migraines. That evening, she again became confused, experienced a 15‐min episode of total aphasia, witnessed by her husband, and had a headache. All symptoms resolved after 3 h. Over the next few months, she continued to have headaches, episodic confusion, and aphasia. Because an aura preceded symptoms, she would stop performing surgery, sit down, or stop driving. Initially, these episodes occurred one to two times a month; however, over the next 4 years, all symptoms increased in frequency and duration, occurring 20–25 days a month and lasting minutes to hours. She subsequently developed seizures and balance and disequilibrium problems causing gait impairment and falls.

She was examined by multiple physicians. An extensive neurological workup included an MRI performed in August 2013; all results were negative except for electroencephalographic “left temporal lobe slowing.” After treatment with the anticonvulsant levetiracetam, she reported a 3‐week period of mental clarity and felt normal. Subsequently, symptoms recurred and she did not respond to dose adjustment or additional medications. Her illness progressively worsened, and she stopped working 4 years after symptom onset.

Based on her clinical history, high arthropod and feral cat exposure risk, ambiguous neurological test results, and failure to improve with medical therapy, a search for an infectious cause was initiated. Initial serology results (Quest Diagnostics, Inc.) were negative for *Borrelia burgdorferi, Anaplasma phagocytophilum, Ehrlichia chaffeensis, Babesia, Bartonella*,* Toxoplasma*, and *Cryptococcus*. In December 2014, a repeat MRI revealed left frontal convexity falx calcifications. A SPECT scan documented heterogeneous decreased uptake bilaterally in the frontal and anterior temporal lobes with greater decrease involving the left hemisphere. Cerebrospinal fluid analyses were within laboratory reference ranges. Due to progressive illness and lack of a diagnosis, immunological testing was performed. There were decreased total IgG and deficiencies in subclasses 1, 2, and 3. CD3, CD4, and CD8 absolute cell counts were abnormally low, and a natural killer cell (NK) function assay was profoundly low (Table 1).

**Table 1:** Selected hematologic and immunological test results for Patients 1 and 2 by month and year (MM/YY)

	Ref Range	Patient 1						Patient 2					
		12/14	12/14	2/15	7/15^a^	10/15	12/15	2/15	4/15	9/15	9/15	10/15	11/15
WBC	3.8–10.8	5.1		6.0	4.9	8.9	6.5	3.6	3.6	4.3		6.0	
HCT	38.5–45.0	38.6		38.4	38.5		39.6	34.4	36.3	37.1		35.3	
PLT	140–400	332		295	273	277	272	327	301	209		244	
ANC	1500–7800	3570		4680	3675	7716	4570	2304	2448	2731		4368	
ALC^b^	850–3900	1275		1020	960	1024	1541	828	828	692		1056	
IGG 1	382–929	287	318	328	415			470	558		507		522
IGG 2	241–700	183	174	176	338			356	398		347		360
IGG 3	22–178	20	20	20	24			20	25		20		23
IGG 4	4–86	5.4	4.1	6.0	12.1			35.4	45.1		62.5		63
Total IgG	694–1618	545	581	560	825	860		881	1054		1005		960
CD3+ ABS	840–3060	1060		811		643	1299	673	664		758		797
CD4+ ABS	490–1740	775		562		395	844	548	547		625		679
CD8+ ABS	180–1170	265		223		219	372	118	125		130		154
Help/ Supp Ratio	0.86–5.00	2.93		2.52		1.81	2.27	4.64	4.39		4.83		4.41
NK Cells ABS	70–760	168		107		251	297	98	95		102		92
CD19+ ABS	110–660	162		64		101	160	56	55		51		55
ALC^c^	850–3900	1380		998		996	1763	840	819		935		952
NK Cells, FUNC	7–125	3				4	10	6	14		14		8

a IVIG administered prior to obtaining blood specimen for this testing date.

b Absolute lymphocyte count derived from Coulter Counter.

c Absolute lymphocyte count derived from flow cytometry.

Ref (reference) range; WBC, white blood cells (10^9^ cells/L); HCT, hematocrit (volume %); PLT, platelet count (10^9^ cells/L); ANC, absolute neutrophil count (10^9^ cells/L); ALC, absolute lymphocyte count (10^9^ cells/L); IgG, immunoglobulin G (g/L); CD3+ ABS, absolute CD3+ cells (10^9^ cells/L); CD4+ ABS, absolute CD4+ cells (10^9^ cells/L); CD8+ ABS, absolute CD8+ cells (10^9^ cells/L); Help/supp (helper/suppressor) ratio; NK cells ABS, absolute natural killer cells (10^9^ cells/L); CD19+ ABS, absolute CD19+ cells (10^9^ cells/L); NK cells, FUNC, functional natural killer cells (LU30). The LU 30 represents the number of lytic sets contained within a preparation of 10 million lymphocytes. One lytic set is defined as the number of lymphocytes required to lyse 30% of the target cells in the NK assay (Quest Diagnostics).

Because immunological test results were abnormal, additional infectious disease testing was pursued. Given her daily work with cats, frequent exposure to fleas, and historical mite infestation, *Bartonella* spp. bacteremia remained a diagnostic consideration. EDTA‐anticoagulated blood specimens were screened for *Bartonella* using the *Bartonella* alpha‐proteobacteria growth medium (BAPGM) enrichment culture/PCR diagnostic platform. (*Bartonella* enrichment PCR™ or ePCR™ (Galaxy Diagnostics, Inc., Research Triangle Park, NC)) Serological analysis of *Bartonella henselae* and *Bartonella quintana* was performed using indirect fluorescent antibody (IFA) testing (Galaxy Diagnostics, Inc., Research Triangle Park, NC). *Bartonella henselae* DNA was amplified and sequenced from a 21‐day BAPGM enrichment blood culture. Serum was not reactive to *B. henselae* or *B. quintana* antigens at 1:16 or 1:32 screening dilutions. Genus PCR assays (Galaxy Diagnostics, Inc., Research Triangle Park, NC) for *Anaplasma, Babesia, Ehrlichia,* and *Rickettsia* spp. were negative.

Based on these results, she was treated with clarithromycin, rifampin, and intravenous gamma globulin every 3 weeks. By week six of antibiotic/IVIG administration, her headaches slowly improved, and she returned to work. After 9 months of antibiotics, she was *B. henselae* and *B. quintana* seroreactive at titers of 1:128 and 1:64, respectively. Three BAPGM enrichment blood cultures collected on alternate days were PCR negative. During the subsequent 9‐month follow‐up, she has experienced no seizures. An MRI, repeated 14 months after the December 2014 MRI study, and 11 months after beginning antibiotics, was unchanged. Her previously abnormal lymphocyte subsets normalized, although NK function remained depressed at 10.

### Patient 2

A 69‐year‐old previously healthy woman hiking in New York State during November 2013 became acutely ill 3 weeks after returning to California. She reported flu‐like symptoms including headaches, nausea, vertigo with disequilibrium, and fatigue but no fever or pain. She developed a bulls‐eye rash on her right infraclavicular fossa, consistent with erythema chronicum migrans. Lyme disease was diagnosed by another physician, who initiated doxycycline treatment for 2 weeks.

She continued to have symptoms and was examined by the primary author 10 days after starting doxycycline (Open Medicine Institute, Mountain View, CA). Laboratory testing results for *Borrelia burgdorferi, Anaplasma phagocytophilum, Ehrlichia chaffeensis, Babesia,* and *Bartonella* antibodies (Quest Diagnostics, Inc.) were negative. Doxycycline was continued for 4 weeks resulting in a transient decrease in symptoms. Headaches, vertigo, disequilibrium, and nausea recurred 2 weeks after completing antibiotics. Treatment with doxycycline and azithromycin initiated on January 2014 resulted in symptomatic improvement; however, antibiotic administration ceased after 2 weeks due to a photosensitivity reaction while visiting Florida. Symptoms returned after 2 weeks, only to resolve again with amoxicillin and azithromycin administration. Within 5 days, she developed severe diarrhea, antibiotics were stopped, and within 3 days, her headache and vertigo returned. A neurology workup in August 2014, including MRI, MRA, and cerebral angiogram, detected no abnormalities.

In October 2014, she was treated with minocycline and metronidazole for headaches, vertigo, and disequilibrium. After 5 weeks, there was symptomatic improvement that continued through 8 weeks, when antibiotics were stopped. Six weeks later, all symptoms recurred. In April 2015, immunological testing documented an IgG subclass 3 deficiency; low absolute CD3, CD8, and CD19 cell counts; and decreased NK cell function (Table 1). Repeat serological testing (Quest Diagnostics, Inc.) for tickborne diseases remained negative. *Bartonella henselae* DNA was amplified and sequenced from a 21‐day BAPGM enrichment blood culture (*Bartonella* enrichment PCR™ or ePCR™ (Galaxy Diagnostics, Inc., Research Triangle Park, NC)). Serum was not *B. henselae* or *B. quintana* seroreactive at 1:16 or 1:32 screening dilutions. Genus PCR assays (Galaxy Diagnostics, Inc., Research Triangle Park, NC) for *Anaplasama, Babesia, Ehrlichia,* and *Rickettsia* spp. were negative. After being treated with clarithromycin and rifampin for 5 months, headaches and vertigo were almost completely resolved and she remained *B. henselae* and *B. quintana* IFA seronegative, although lymphocyte subsets and NK function remained abnormal. She remained *B. henselae* or *B. quintana* seronegative, and three BAPGM enrichment blood cultures collected on alternate days were PCR negative.

## Discussion

Two important clinical observations evolved out of the microbiological, immunological, and therapeutic findings associated with the medical management of these two patients. First, persistent or recurrent neurological symptoms of undetermined etiology in patients with historical vector exposures should prompt testing for bartonellosis. Historically, *B. henselae* infections in immunocompetent individuals have been associated with self‐limiting cat scratch disease, whereas recent research supports persistent and potentially relapsing bacteremia 1, 2, 3. As previously reported 2, 3, both of these *B. henselae* bacteremic patients experienced headaches and disequilibrium. Patient 1 also experienced seizures, episodic confusion, and aphasia, which resolved completely following antibiotic therapy, despite persistence of the MRI abnormalities 1. Secondly, immunological testing should be concurrently pursued to determine whether defective immune function accompanies neurological symptoms. Both patients had immunological abnormalities, including suppression of NK function, despite lacking a prior medical history indicative of immunodeficiency. The extent to which persistent *B. henselae* bacteremia may have induced immunocompromise or whether a chronic latent infection resulted in bacterial reactivation is unknown. It is important to note that after 9 months of treatment, the CD3, CD4, and CD19 deficiencies in Patient 1 resolved, while Patient 2 remained immunologically impaired after 5 months of therapy. There remains a substantial need for sequential electroencephalographic, MRI, immunological, and bacteriological patient data to guide physician decision making in patients with longstanding *B. henselae* bacteremia.

Using a previously validated diagnostic approach 3, 4, *B. henselae* bacteremia was confirmed in both patients by BAPGM enrichment blood culture, PCR amplification, and DNA sequence confirmation. Importantly, PCR did not amplify *B. henselae* DNA from patient's blood, serum, 8‐day, or 14‐day BAPGM enrichment blood cultures, supporting the need for prolonged bacterial incubation times to obtain PCR confirmation for some *B. henselae* bacteremic patients. Consistent with previous studies^3^, ^5^ in which a subset of patients with persistent bacteremia were not IFA seroreactive to a panel of *Bartonella* sp. antigens, neither patient was initially *B. henselae* or *B. quintana* IFA seroreactive, whereas antibody reactivity was documented after antibiotic treatment in Patient 1, potentially due to enhanced immunological recognition of antigenic epitopes. Based upon these patients and previously published studies 3, 5, enrichment blood culture and PCR should be used in conjunction with *Bartonella* sp. serological analysis when attempting to confirm bacteremic infection with a *Bartonella* sp.

Vector transmission of *B. henselae* was the suspected source of infection for both patients. The veterinarian had ongoing vector (flea, mite, and potentially ticks) and animal exposure, which are occupational risks for animal health workers 3, 5. The cat flea (*Ctenocephalides felis*) transmits *B. henselae* among cats that develop a relapsing bacteremia and remain persistently infected reservoir hosts for months to years 6. Although there is no evidence that cat‐associated mites (*S. scabiei* or *N. cati*) are vector competent for the transmission of *Bartonella* species, rat mite (*Ornithonyssus bacoti*) and pigeon mite (*Dermanyssus* spp.) transmissions of *B. henselae* and *B. quintana,* respectively, have been suspected 7, 8. Due to an acute‐onset illness and presumed tick attachment in a Lyme‐endemic region with the subsequent development of erythema chronicum migrans, Lyme disease was initially suspected in Patient 2. Rapid treatment with doxycycline may have prevented serodiagnostic confirmation of *B. burgdorferi* transmission, whereas testing for other tickborne pathogens was negative. Although tick transmission of *B. henselae* has not been proven, organism‐specific DNA has been PCR‐amplified and sequenced from *Ixodes* sp. ticks 9, vector competence for *Bartonella* transmission has been demonstrated in a rodent model 10, and French investigators have recently documented *Bartonella* spp. bacteremia in patients following tick exposures 11.

Historically, systemic bartonellosis has been reported in immunocompromised patients, such as those with HIV/AIDS and transplant recipients. Recently, infection with *Bartonella* spp. has been reported in healthy asymptomatic Brazilian blood donor candidates^12^ and in previously immunocompetent patients with chronic neurological or rheumatologic symptoms 2, 3, 5.

Because *Bartonella* spp. can infect erythrocytes, endothelial cells, and various macrophage‐type cells, including brain‐derived dendritic cells in vitro, the spectrum of neurological symptoms attributable to bartonellosis appear to be extremely diverse among patients 1, 2. Physicians should be aware of the rapidly increasing number of *Bartonella* spp., the large number of proven and suspected arthropod vectors, and the large number of reservoir hosts, all of which are collectively contributing to the enhanced recognition of neurobartonellosis as a medically important emerging infectious disease.

## Authorship

DK: principal investigator and physician of patients presented in this publication – analyzed clinical data and prepared the manuscript. AMK: provided medical consultation and received funding resources. RBM: provided medical consultation. NAC: involved in laboratory data collection and analysis and prepared the manuscript. EBB, MD: principal investigator – is involved in data analysis and manuscript preparation.

## Conflict of Interest

In conjunction with Dr. Sushama Sontakke and North Carolina State University, Edward B. Breitschwerdt, DVM, holds U.S. Patent No. 7,115,385; Media and Methods for cultivation of microorganisms, which was issued on 3 October 2006. He is a co‐founder, shareholder, and chief scientific officer for Galaxy Diagnostics, a company that provides advanced diagnostic testing for the detection of *Bartonella* species infections. Robert B. Mozayeni, MD, is chief medical officer, and Natalie Cherry, PhD, is the laboratory supervisor and research analyst for Galaxy Diagnostics. The remaining authors have no competing interests.
